# Supplementary material for: Bumblebees increase their learning flight altitude in dense environments
Source: J Exp Biol. 2025 May 12;228(9):jeb249714. doi: 10.1242/jeb.249714 (PMC12091867; doi:10.1242/jeb.249714)
Supplement: Supplementary information [file jexbio-228-249714-s1.pdf]

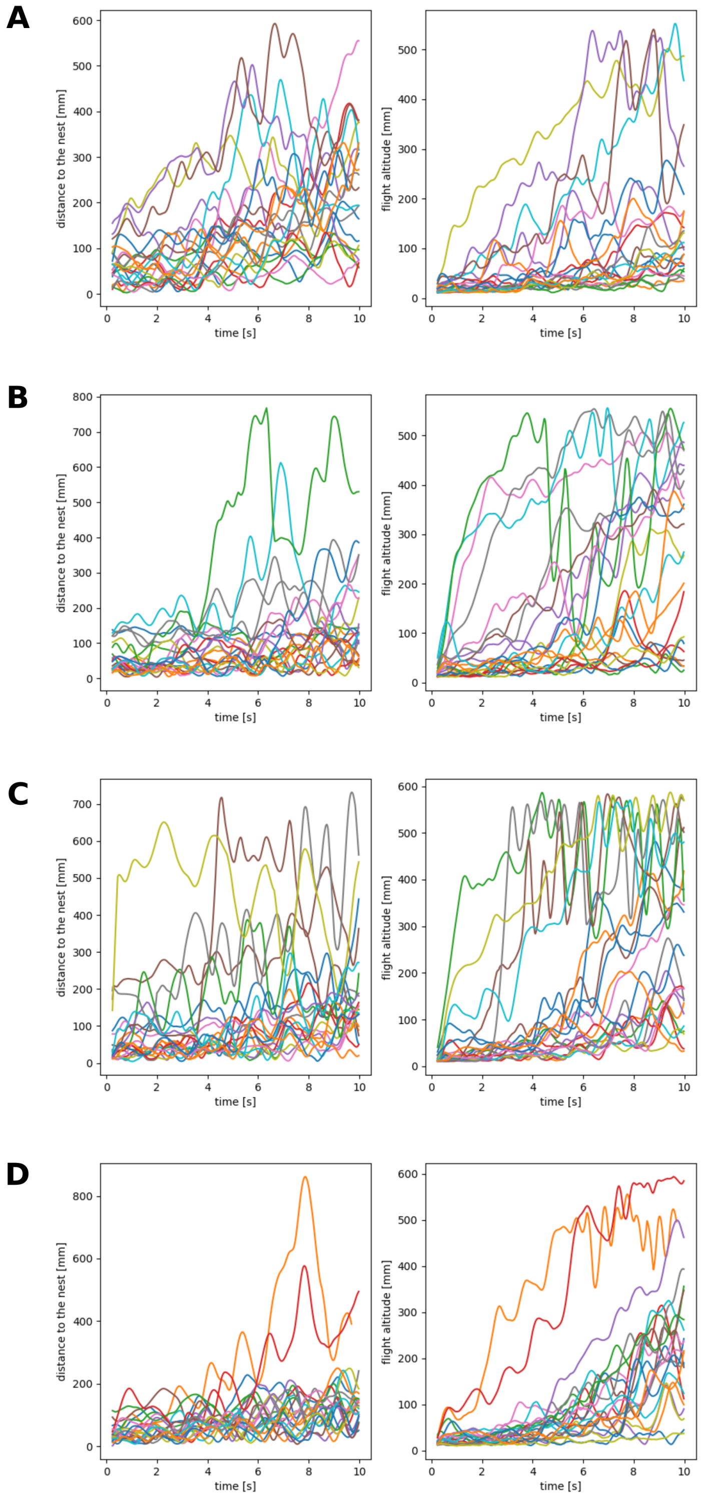

Fig. S1. **Distance to the nest and flight altitude.** Distance to the nest and height for each learning flight (z 0.01m) over time within the time window of 10 seconds after take-off for the four test conditions (A: three objects, B: full-density, C: half-density, D: outer ring).

Journal of Experimental Biology • Supplementary information

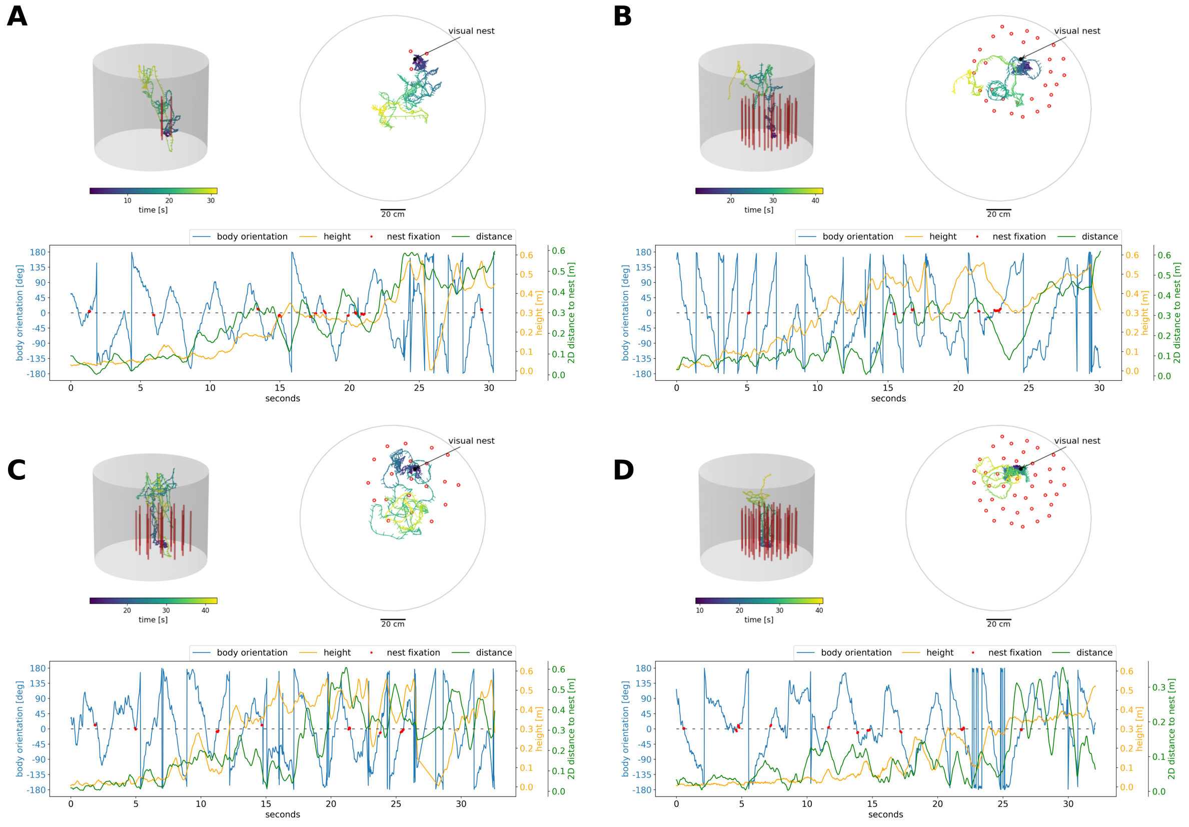

Fig. S2. **Exemplary trajectories.** Exemplary trajectories in 3D and 2D (top view) in the four tested environments (A, three objects, B: half-density, C: outer ring and D: full-density) with the body orientation, height and 2D distance to the nest for the initial walking phase and the first 30 seconds of flight. The fixations of the nest are highlighted in red.

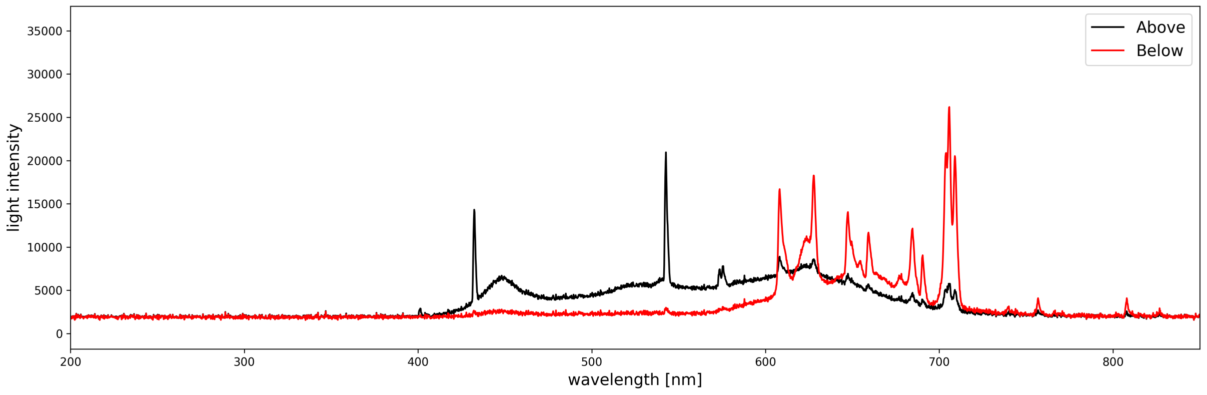

Fig. S3. **Light spectrum within the flight arena.** The light spectrum recorded within the flight arena from above (black) and from below (red). The light from above the arena ranges between 400 and 700 nm which is within the range of visible light to humans. The light from below, used for tracking the bee position, ranges between 600 and 700nm which is outside the perception of bees Skorupski, D¨oring, and Chittka (2007).

Table S1. Number of bees from colonies 1-3 in the four tested environments.

hive	three objects	outer ring	half-density	full-density
1	17	0	0	19
2	5	12	12	3
3	0	10	10	0
total	22	22	22	22

Table S2. Results of the Rayleigh test for circular uniformly distributed data of the body orientation of the bees in all four environments and spatial categories. The statistical results of the z-value, p-value and significance level are given for each spatial area and the tested environmental condition.

condition	area	z	p-value	significance level
Three objects	low clutter	389.684	1.38e-170	***
	high clutter	609.359	1.83e-270	***
	above clutter	825.417	0	***
	below outside	356.904	2.85e-16	***
	above outside	137.680	1.03e-06	***
full-density	low clutter	221.989	2.42e-97	***
	high clutter	661.197	1.53e-29	***
	above clutter	123.114	3.01e-54	***
	below outside	548.840	1.16e-24	***
	above outside	182.583	1.77e-82	***
half-density	low clutter	770.239	0	***
	high clutter	247.969	1.65e-11	***
	above clutter	570.329	1.61e-249	***
	below outside	212.517	0.119	ns
	above outside	900.812	0.0001	**
Outer ring	low clutter	287.988	4.40e-126	***
	high clutter	296.394	1.29e-13	***
	above clutter	190.498	1.45e-83	***
	below outside	188.734	6.26e-09	***
	above outside	148.903	3.34e-07	***

Table S3. The detailed statistical results of the Kuiper test and the v-test can be found in a separate file

Available for download at
https://journals.biologists.com/jeb/article-lookup/doi/10.1242/jeb.249714#supplementary-data

References

Skorupski, P., T. F. Döring, and L. Chittka (2007). "Photoreceptor spectral sensitivity in island and mainland populations of the bumblebee, Bombus terrestris". In: *Journal of Comparative Physiology A* 193.5, pp. 485–494. DOI: 10.1007/s00359-006-0206-6.

Journal of Experimental Biology • Supplementary information
